# Aberrant epigenetic regulation of GABRP associates with aggressive phenotype of ovarian cancer

**DOI:** 10.1038/emm.2017.62

**Published:** 2017-05-19

**Authors:** Hye Youn Sung, San-Duk Yang, Woong Ju, Jung-Hyuck Ahn

**Affiliations:** 1 Department of Biochemistry, School of Medicine, Ewha Womans University, Seoul, Korea; 2 Department of Biomedical Sciences, Seoul National University, College of Medicine, Seoul, Korea; 3 Department of Obstetrics and Gynecology, School of Medicine, Ewha Womans University, Seoul, Korea

## Abstract

Metastasis is a major cause of therapeutic failure in ovarian cancer. To elucidate molecular mechanisms of ovarian cancer metastasis, we previously established a metastatic xenograft mouse model using human ovarian carcinoma SK-OV-3 cells. Using gene expression profiling, we found that γ-aminobutyric acid (GABA)_A_ receptor π subunit (*GABRP*) expression was upregulated (>4-fold) in metastatic tissues from our xenograft mice compared with SK-OV-3 cells. Importantly, *GABRP* knockdown diminished the migration and invasion of SK-OV-3 cells, and reduced extracellular signal-regulated kinase (ERK) activation while overexpression of *GABRP* exhibited significantly increased cell migration, invasion and ERK activation. Moreover, treatment with the mitogen-activated protein kinase (MAPK)/ERK kinase (MEK) inhibitor U0126 similarly suppressed the migration and invasion of SK-OV-3 cells, implying that GABRP promotes these cellular behaviors by activating the MAPK/ERK pathway. Using genome-wide DNA methylation profiling, we identified hypomethylated CpG sites in the *GABRP* promoter in metastatic tissues from the xenograft mice compared with SK-OV-3 cells. Treatment with a DNA methyltransferase inhibitor demonstrated that methylation at −963 bp from the *GABRP* transcription start site (−963 CpG site) was critical for the epigenetic regulation of *GABRP*. Finally, we analyzed human ovarian cancer patient samples and showed DNA hypomethylation at the *GABRP* −963 CpG site in advanced stage, but not early-stage, primary tumors compared with their paired normal tissues. These findings suggest that GABRP enhances the aggressive phenotype of ovarian cancer cells, and that the DNA methylation status of the *GABRP* −963 CpG site may be useful for predicting the metastatic potential in ovarian cancer patients.

## Introduction

Ovarian cancer has the highest mortality rate among gynecological cancers, with a 5-year survival rate of only 27% when the disease is diagnosed at an advanced stage.^1^ The majority of ovarian cancer patients are diagnosed at an advanced stage because they show few clear symptoms, and there are no reliable diagnostic tools for early detection. Ovarian cancer is unique in that it mainly metastasizes via a transcoelomic route leading to ascites-mediated intraperitoneal dissemination rather than hematogenous dissemination.^2^ The intraperitoneal dissemination occurs relatively early in the disease, which is another reason why ovarian cancer is rarely diagnosed at an early stage.^3^ Because metastasis prevention is the major obstacle in the clinical management of ovarian cancer, a comprehensive understanding of the molecular mechanisms that regulate the aggressive behavior of ovarian cancer cells is required to improve treatment outcomes for ovarian cancer patients.

We previously injected human ovarian carcinoma SK-OV-3 cells into the peritoneum of female nude mice to establish an ovarian cancer xenograft mouse model.^4^ We then performed expression profiling to identify genes that were differentially expressed in the metastatic tissues, compared with the injected SK-OV-3 cells, from the xenograft mice.^4, 5, 6, 7^ Among the genes whose expression was altered more than twofold in metastatic tissues, we selected γ-aminobutyric acid (GABA)_A_ receptor π subunit (*GABRP*), which was upregulated in the metastatic tissue, for further study.

GABA is the major inhibitory neurotransmitter in the mature mammalian central nervous system; it functions through activation of the GABA receptors, which include the ionotropic (GABA_A_ or GABA_C_) and the metabotropic (GABA_B_)^8, 9^ receptors. The GABA_A_ receptor is a heteropentameric, ligand-gated chloride channel comprised of various subunits (α1–6, β1–3, γ1–3, δ, ɛ, θ, π and ρ1–3).^10, 11^

GABA and GABA receptors are also expressed in peripheral non-neuronal cells, suggesting that they have additional functions beyond regulating neurotransmission. GABA and GABA receptors have been reported to regulate proliferation, differentiation, migration and development of many peripheral non-neuronal cells; however, their precise roles in non-neuronal cells, including cancer cells, are largely unknown.^12^ Conversely, GABRP is mainly expressed in non-neuronal tissues, including the mammary gland, prostate gland, lung, thymus and uterus; it is not found abundantly in the brain.^13, 14^ GABRP is also highly expressed in certain types of cancer such as pancreatic ductal adenocarcinoma^15^ and basal-like breast cancer.^16^ In pancreatic ductal adenocarcinoma, GABRP promotes proliferation by increasing intracellular Ca^2+^ levels and activating the mitogen-activated protein kinase/extracellular signal-regulated kinase (MAPK/ERK) signaling pathway.^15^ In basal-like breast cancer cells, GABRP stimulates the migratory activity and expression of basal-like cytokeratins through ERK cascade.^16^

On the basis of these previously established roles of GABRP in tumorigenesis and our identification of *GABRP* as an upregulated gene in metastatic tissues compared with ovarian carcinoma cells from our xenograft mouse model, we hypothesized that high GABRP expression in ovarian carcinoma cells actively enhances their metastatic potential. Thus, in this study, we performed *GABRP* gain- and loss-of-function studies in the SK-OV-3 ovarian carcinoma cell line to investigate its role in ovarian cancer metastasis. Moreover, we analyzed the DNA methylation status of the *GABRP* promoter using our previously established ovarian cancer xenograft mouse model to determine whether the differential expression of *GABRP* in metastatic tissues is epigenetically regulated. Finally, we analyzed ovarian cancer patient samples to determine whether *GABRP* expression and/or epigenetic modifications are clinically relevant.

## Materials and methods

### Cell culture

The human ovarian cancer cell line SK-OV-3 was purchased from American Type Culture Collection (ATCC, no. HTB-77, Manassas, VA, USA) and cultured in McCoy's 5A medium (Gibco/BRL, Rockville, MD, USA) supplemented with 10% fetal bovine serum (FBS, Gibco/BRL), 100 U ml^−1^ penicillin (Gibco/BRL) and 100 μg ml^−1^ streptomycin (Gibco/BRL) in an atmosphere of 95% humidified air and 5% CO_2_ at 37 °C.

### Ovarian cancer xenograft mouse model

All procedures for handling and killing the animals used in this study were performed in strict compliance with the guidelines of the Korean animal protection law, and were approved by the Institutional Animal Care and Use Committee (IACUC) of Ewha Womans University School of Medicine. SK-OV-3 cells (2 × 10^6^) suspended in culture medium were injected intraperitoneally into 10 female nude mice (CAnN.Cg-Foxn1^NU^, 4–6 weeks old). Four weeks after inoculation, the xenograft mice were killed, and at least four tumor metastases adhering to the mesothelial surface of the peritoneum were harvested from each mouse.

### Processing of mRNA microarray and gene expression analysis

Total RNA was extracted from the tumor metastases of the mice and SK-OV-3 cells using the RNeasy Mini Kit (Qiagen, Valencia, CA, USA). One microgram of total RNA was amplified and labeled according to the Affymetrix GeneChip Whole Transcript Sense Target Labeling protocol. The labeled cDNA was hybridized to Affymetrix Human Gene 1.0 ST arrays (Affymetrix, Santa Clara, CA, USA). The scanned raw expression values were background-corrected, normalized and summarized using the Robust Multiarray Averaging approach in the Bioconductor ‘affy' package (Affymetrix). The resulting log_2_-transformed data were used for further analyses.

To identify differentially expressed genes (DEGs), we applied moderated t-statistics based on an empirical Bayesian approach.^17^ Significantly upregulated and downregulated DEGs were defined as genes with at least a twofold difference in expression levels between the xenograft tissues and SK-OV-3 cells after multiple testing correction (Benjamini–Hochberg false-discovery rate-adjusted *P*-value <0.05).^18^ Finally, we excluded genes with a low expression level (maximum log_2_ expression level in a total of eight samples <7.0) from the list of DEGs.

### RNA preparation and reverse-transcription quantitative polymerase chain reaction

Total RNA was extracted from the tumor metastases and SK-OV-3 cells using the RNeasy Mini Kit (Qiagen) according to the manufacturer's protocol. One microgram of total RNA was converted to cDNA using Superscript II reverse transcriptase (Invitrogen, Carlsbad, CA, USA) and oligo-(dT)_12–18_ primers (Invitrogen) according to the manufacturer's instructions. Reverse-transcription quantitative polymerase chain reaction (RT-qPCR) was performed in a 20 μl reaction mixture containing 1 μl cDNA, 10 μl SYBR Premix EX Taq (Takara Bio, Otsu, Japan), 0.4 μl Rox reference dye (50 ×, Takara Bio), and 200 nM primers for each gene. The primer sequences were: *GABRP* (forward), 5′-CTCGATTCAGTCCCTGCAAGA-3′ *GABRP* (reverse), 5′-GTGCGGGACCCGATCAT-3′ *GAPDH* (forward), 5′-AATCCCATCACCATCTTCCA-3′ and *GAPDH* (reverse), 5′-TGGACTCCACGACGTACTCA-3′. The reactions were run on a 7500 Fast Real-Time PCR System (Applied Biosystems, Foster City, CA, USA) at 95 °C for 30 s, followed by 40 cycles of 95 °C for 3 s and 60 °C for 30 s and a single cycle of 95 °C for 15 s, 60 °C for 60 s, and 95 °C for 15 s to generate dissociation curves. All PCR reactions were performed in triplicate, and the specificity of the reaction was determined by melting curve analysis. Comparative quantification of each target gene was performed based on cycle threshold (C_t_) normalized to *GAPDH* using the ΔΔC_t_ method.

### Genomic DNA isolation and CpG methylation microarray

Genomic DNA was extracted from cell lines and tumor metastases using a QIAmp mini kit (Qiagen) according to the manufacturer's instructions. The Illumina HumanMethylation450 BeadChip (Illumina, San Diego, CA, USA) that targets ~450 000 CpG sites was used for genome-wide screening of DNA methylation. DNA methylation values were described as *β*-values, which were calculated by subtracting the background using negative controls on the array and taking the ratio of the methylated signal intensity against the sum of both methylated and unmethylated signals. The *β*-values ranged from 0 (completely unmethylated) to 1 (fully methylated) on a continuous scale for each CpG site. To identify differentially methylated CpG sites, we applied the difference in mean *β*-values (Δ*β*; mean *β*-value in tumors−mean *β*-value in SK-OV-3). If the absolute difference of the mean *β*-values (|Δ*β*|) was >0.2, the site was defined as a differentially methylated CpG site. We described a CpG site as hypermethylated if Δ*β* was greater than 0.2 and hypomethylated if Δ*β* was less than −0.2.

### Bisulfite sequencing PCR

Genomic DNA was extracted from the harvested tumor metastases of the ovarian cancer mouse xenografts and SK-OV-3 cells using the QIAmp DNA mini kit (Qiagen) according to the manufacturer's protocol. Bisulfite treatment of the genomic DNA was performed using the EpiTect Bisulfite Kit (Qiagen) according to the manufacturer's instructions. For bisulfite sequencing of the target promoter region of *GABRP*, bisulfite sequencing PCR (BSP) was carried out using conventional PCR in a 50 μl reaction mixture containing 10 ng bisulfite-modified genomic DNA, 1.5 mM MgCl_2_, 200 μM dNTP, 1 U Platinum Taq polymerase (Invitrogen), 1 × Platinum Taq buffer and 200 nM gene-specific BSP forward and reverse primers. The BSP primers were designed using MethPrimer software (http://www.urogene.org/methprimer). For *GABRP*, the BSP product was 452 bp (position in the human GRCh37/hg19 assembly: chromosome 5, 170209520–170209971) and contained five CpG sites. The five CpG sites are located at −1178, −1142, −994, −963 and −924 from the transcription start site. The BSP primer sequences were: (forward), 5′-TGAGTTTTTTTAGGAGAAATGAAAG-3′ and (reverse), 5′-CTAAACTCTAAATCATCCCCTCATC-3'. The reaction ran at 95 °C for 5 min, followed by 30 cycles of 95 °C for 30 s, 55 °C for 30 s and 72 °C for 30 s, and a final elongation step at 72 °C for 5 min.

The BSP products were purified using the QIAquick Gel Extraction kit (Qiagen) according to the manufacturer's protocols and ligated into the yT&A cloning vector (Yeastern Biotech, Taipei, Taiwan). The ligation products were used to transform competent DH5α *Escherichia coli* cells (RBC Bioscience, Taipei, Taiwan) using standard procedures. Blue/white screening was used to select bacterial clones, and BSP product-positive clones were confirmed by colony PCR using the BSP primers to verify the insert size. Plasmid DNA was then extracted from at least 15 insert-positive clones using the QIAprep Spin Miniprep kit (Qiagen) and sequenced using the M13 primer to analyze the methylation status at specific CpG sites.

### Quantitative methylation-specific PCR

Quantitative methylation-specific PCR (qMSP) was carried out with bisulfite-modified genomic DNA as the template and specific primer sequences designed to detect the methylated and unmethylated forms of two CpGs at −1142 and −963 bp from the transcription start site. The following methylated/unmethylated-specific primers were used: −1142 CpG (methylated forward), 5′-GTTTTTTTAGGTGTTTATTTTTTATTTATTAGC-3′ −1142 CpG (unmethylated forward), 5′-GTTTTTTTAGGTGTTTATTTTTTATTTATTAGT-3′ −1142 CpG (reverse), 5′-AAACTCCTACCTAAACCCAATCT-3′, −963 CpG (methylated forward), 5′-GATTGGGTTTAGGTAGGAGTTTC-3′ −963 CpG (unmethylated forward), 5′-GATTGGGTTTAGGTAGGAGTTTT-3′ and −963 CpG (reverse), 5′-CAAACTCACTCTTAACTCCAAC-3′. For qMSP, a 20 μl reaction mixture containing 2 μl (10–100 ng μl^−1^) bisulfite-treated DNA, 10 μl SYBR Premix EX Taq (Takara Bio), 0.4 μl Rox reference dye (50 × ; Takara Bio), and 200 nM each primer was amplified using a 7500 Fast Real-Time PCR system (Applied Biosystems). The amplification reaction conditions were 95 °C for 30 s, followed by 40 cycles of 95 °C for 3 s and 63 °C for 30 s. The PCR product was then cycled at 95 °C for 15 s, 60 °C for 1 min, and 95 °C for 15 s to generate the dissociation curves for analysis. The methylation percentage at each CpG site was calculated as follows (C_t_ represents the threshold cycle):


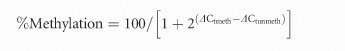


### 5-aza-2′-deoxycytidine treatment

To demethylate methylated CpG sites, SK-OV-3 cells were treated with an increasing concentration (0, 5, and 10 μM) of 5-aza-2′-deoxycytidine (5-aza-dC; Sigma-Aldrich, St Louis, MO, USA) for 3 days. The culture medium was replaced daily.

### Transient transfection

To establish a transient expression system, SK-OV-3 cells were transfected with pCMV6-GABRP (Origene, Rockville, MD, USA) or pEGFP-N3 (Clontech, Mountain View, CA, USA) plasmids using Lipofectamine 2000 (Invitrogen). Briefly, cells were plated at 6 × 10^5^ cells per well in six-well plates and allowed to grow overnight. Two micrograms of each plasmid and 5 μl of Lipofectamine 2000 were diluted in Opti-MEM medium (Gibco/BRL) to a total volume of 250 μl. The plasmid-Lipofectamine 2000 mixture was incubated at room temperature for 20 min. The transfection mixture was added to the six-well plates containing complete growth medium, and plates were incubated at 37 °C for 24 h in a 5% CO_2_ incubator. Overexpression of *GABRP* was confirmed using qPCR 24 h post transfection.

Pre-designed small interfering RNA (siRNA) for *GABRP* (siGABRP, CAT#ID LU-006176-00-0005) and a non-targeting control (siNC, CAT#ID D-001206-13-05) were purchased from GE Healthcare (Lafayette, CO, USA). To deplete *GABRP*, SK-OV-3 cells were transfected with 100 nM siGABRP or siNC using DharmaFECT 1 transfection reagent (GE Healthcare) according to the manufacturer's protocol. Knockdown of *GABRP* was confirmed using RT-qPCR 24 h post transfection.

### Transwell migration and *in vitro* invasion assay

Complete growth medium was changed to serum-free medium 24 h post transfection. After 24 h of serum deprivation, cell migration assays were performed in 24-well transwell plates with 8.0-μm pore size membrane inserts (Corning, New York, NY, USA) as previously described.^4^ SK-OV-3 cells in serum-free medium, with or without the MAPK/ERK kinase (MEK) inhibitor U0126 (10 μM) (Cell Signaling Technology, Danvers, MA, USA), were added at 2 × 10^5^ cells per well into the upper chambers of the inserts, whereas medium containing 15% FBS was added to the lower chambers as a chemoattractant. For each experiment, both chemotactic migration to medium containing 15% FBS and random migration in serum-free medium were assessed in parallel transwell plates for 6 h at 37 °C in a 5% CO_2_ incubator.

The *in vitro* invasion assays were performed using the BD BioCoat Matrigel Invasion Chamber kit (Becton-Dickinson, Franklin Lakes, NJ, USA) as previously described.^4^ SK-OV-3 cells in serum-free medium, with or without 10 μM U0126 (Cell Signaling Technology), were seeded at 1 × 10^5^ cells per well into the upper chambers, whereas the medium containing 10% FBS was added to the lower chambers. The migration through the Matrigel chamber was allowed to proceed at 37 °C for 24 h in a 5% CO_2_ incubator.

After the incubation periods for cellular migration or invasion, the SK-OV-3 cells on the upper side of the filter that had not migrated or invaded were carefully scraped away with cotton swabs, whereas those on the lower side were fixed for 2 min using the Diff-Quick kit solution (Thermo Fisher Scientific, Waltham, MA, USA), stained with 1% crystal violet for 2 min, and washed twice with distilled water at room temperature. The images of stained cells were acquired at × 200 magnification from six different fields. For quantitative analysis, the stained cells were subsequently extracted with 10% acetic acid, and colorimetric measurements were performed at 590 nm.

### Western blot analyses

SK-OV-3 cell lines were lysed in RIPA buffer (Sigma-Aldrich) containing proteinase inhibitor cocktail (Roche Applied Science, Mannheim, Germany). Proteins (20 μg) were resolved using denaturing 10% sodium dodecyl sulfate-polyacrylamide gel electrophoresis and transferred to polyvinylidene fluoride membranes. Membranes were blocked in 5% skim milk in Tris-buffered saline with 0.1% Tween 20 (TBST) and subsequently incubated overnight at 4 °C with the following primary antibodies: p44/42 MAPK (Erk1/2) (137F5) rabbit mAb (1:2000, Cell Signaling Technology), phospho-p44/42 MAPK (Erk1/2) (Thr202/Tyr204) (D13.14.4E) XP rabbit mAb (1:1000, Cell Signaling Technology), and an anti-β-actin antibody produced in rabbit (1:5000, Sigma-Aldrich). After washing, the membranes were incubated with secondary antibodies conjugated to horseradish peroxidase for 1 h at room temperature. Protein bands were detected via chemiluminescence using West Save Star (Ab Frontier, Seoul, Korea) according to the manufacturer's protocol. Bands were visualized using a Luminescent Image analyser LAS-300 (GE Healthcare) and quantified using Image Gauge software (Fuji Photo Film, Tokyo, Japan).

### Tissue specimen collection

Six patients with early stage (stages I and II), and nine patients with advanced stage, ovarian cancer were included in this study. Primary solid tumor and adjacent normal tissues were obtained from the Ewha Biospecimen Bank. Patient information, including age, International Federation of Gynecology and Obstetrics (FIGO) stage, histological grade and histology, was collected. The clinicopathological characteristics are summarized in Table 1. All the experiments were approved by the ethics committee of the Ewha Womans University Medical Center and the Korea National Institute of Health (Permit Number: EUMC 2014-05-004-001), and all the patients had given written informed consent.

### Statistical analysis

Data are expressed as mean±s.d. of at least three independent experiments. Statistical analyses were carried out using GraphPad Prism 5 software (GraphPad, La Jolla, CA, USA), and the details of the statistical analysis for each data set are included in the figure legends. *P*-values <0.05 were considered statistically significant.

## Results

### *GABRP* was increased in the metastatic tissues of ovarian cancer xenograft mice

**Figure 1:**
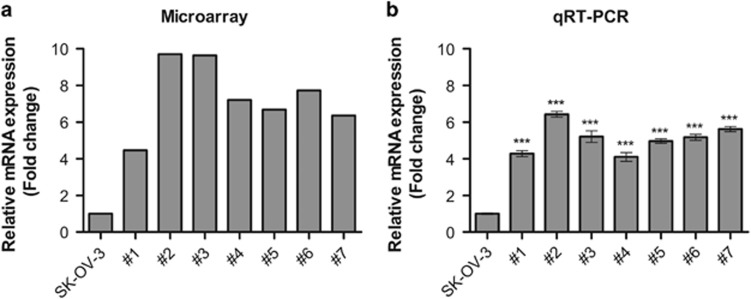
*GABRP* was upregulated in the metastatic tissues of mouse ovarian cancer xenografts. *GABRP* expression levels were determined by expression microarray (**a**) and RT-qPCR (**b**). The error bars indicate the means±s.d.'s of three independent experiments. Statistical analyses were performed using one-way analysis of variance (ANOVA), followed by Tukey's multiple comparison post tests (***P<0.001). Seven pools of metastatic tissues from each individual mouse xenograft are labeled #1–#7 (*n*=7). Three replicate cultures from the different stock of SK-OV-3 cells were conducted (*n*=3).

Previously, we established an ovarian cancer xenograft mouse model by injecting SK-OV-3 cells into the peritoneum of 10 female nude mice. We performed global gene expression profiling and found that 444 genes were downregulated and 529 were upregulated in metastatic tissues compared with SK-OV-3 cells from xenograft mice using the following cut-off criteria: a fold change ⩾2, transcript number >7 and *P*⩽0.05.^4, 5, 6, 7^ Interestingly, the expression levels of *GABRP* were increased ~4.47–9.71-fold in metastatic tissues from xenograft mice compared with those in SK-OV-3 cells (Figure 1a). Consistent with the microarray analysis, we performed RT-qPCR and showed that *GABRP* levels were increased 4.28–6.43-fold in metastatic tissues compared with those in SK-OV-3 cells (Figure 1b).

### *GABRP* significantly enhances ovarian carcinoma cell migration and invasion

**Figure 2:**
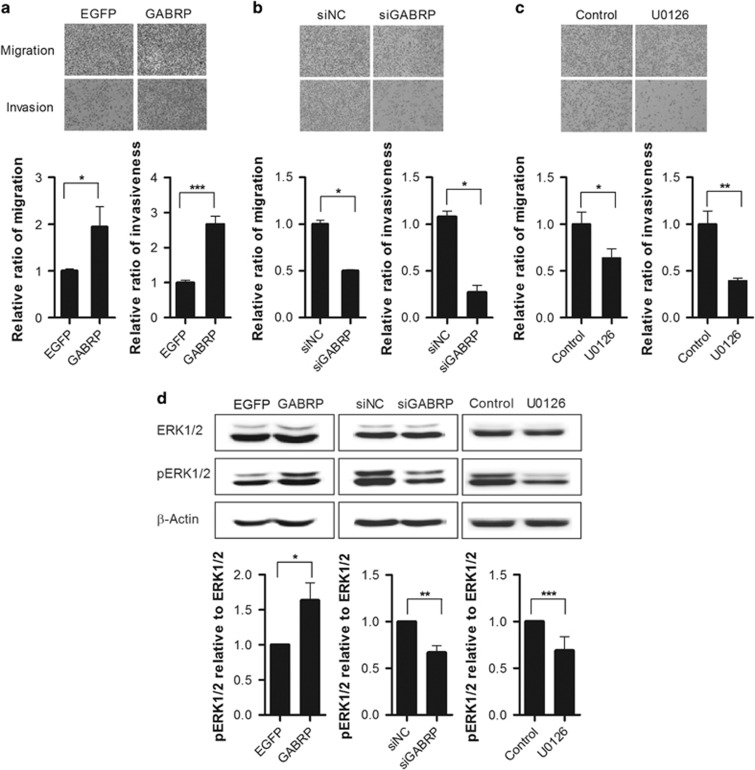
*GABRP* promotes migration and invasion of SK-OV-3 cells through ERK pathway. SK-OV-3 cells were transfected with *EGFP* and *GABRP* expression constructs (**a**) or with siNC and siGABRP (**b**). After transfection, the effects on migration were determined using transwell assays, and the effects on invasion were determined using Matrigel-based invasion assays. Representative images of migrated or invaded cells are shown (× 100 magnification). The amounts of migrated or invaded cells were quantified by measuring the absorbance of extracts from cell stains at 595 nm (**a**, **b**). After treatment with the MEK inhibitor U0129, the migration and invasion of SK-OV-3 cells were determined using transwell and Matrigel-based invasion assays, respectively (**c**). Immunoblot analysis of total ERK1/2 and phosphorylated ERK1/2 proteins was performed in cells treated with expression plasmid constructs, siRNAs or MEK inhibitor (**d**). A representative immunoblot of total ERK1/2 and phosphorylated ERK1/2 proteins (upper panel of **d**), and quantification of phosphorylated ERK1/2 relative to total ERK1/2 (lower panel of **d**), are shown. All data shown are means±s.d.'s for triplicate measurements. Statistical analysis was performed with *t*-tests (*P<0.05, ^**^P<0.01, ^***^P<0.001). siGABRP, *GABRP* siRNA; siNC, non-targeting control siRNA.

To elucidate the functional role of GABRP in metastasis of ovarian cancer, *GABRP* expression plasmid construct was transiently transfected in SK-OV-3 cells. In contrast, endogenous *GABRP* expression was knocked down by transfecting siRNA (siGABRP). After 24 h transfection, endogenous *GABRP* expression was confirmed by RT-qPCR (Supplementary Figure S1). The metastatic potential of the transfected SK-OV-3 cells were detected by transwell migration assay and Matrigel-based invasion assay. The migratory ability and invasiveness of the cells were measured by crystal violet staining of cells that migrated through the membranes. The transwell migration assay showed that overexpression of *GABRP* approximately twofold increased cell migration compared to *EGFP*-transfected cells. In addition to cell migration, the overexpression of *GABRP* was also significantly (~2.68-fold) enhanced invasion of cells through Matrigel (Figure 2a). As expected, the migratory ability of the siGABRP SK-OV-3 cells was decreased by 50% compared with that of siNC-transfected cells. Moreover, the ability of the siGABRP cells to invade through Matrigel was diminished by 73% compared with that of the siNC cells (Figure 2b). These results indicate that GABRP plays a pivotal role in promoting the metastasis of ovarian carcinoma cells.

A recent study reported that GABRP was required for maintaining the pro-migratory behavior of basal-like breast cancer cells via activation of the ERK signaling pathway.^16^ Therefore, we investigated whether the ERK signaling pathway is involved downstream of GABRP in promoting the aggressive behavior of ovarian cancer cells. Indeed, SK-OV-3 cells treated with the MAPK/ERK kinase (MEK) inhibitor U0126 showed a 64% reduction in migration, and a 39% reduction in invasion, compared with those of untreated cells (Figure 2c). Furthermore, immunoblot analyses confirmed that ERK1/2 activation was effectively inhibited by U0126 and showed that overexpression of *GABRP* enhanced ERK1/2 activation while *GABRP* knockdown diminished ERK1/2 activation (Figure 2d) indicating that GABRP promotes the metastatic potential of ovarian carcinoma cells through ERK pathway activation. Next, we examined whether alteration of *GABRP* expression could affect intracellular calcium signaling. SK-OV-3 cells were transfected with *EGFP* and *GABRP* expression constructs or with siNC and siGABRP. After 24 h transfection, the cells were treated with 100 μM muscimol, a GABA_A_ receptor agonist. Intracellular calcium concentration was determined using a colorimetric calcium detection kit. Although muscimol treatment increased intracellular calcium concentrations by 1.96-fold, overexpression of *GABRP* did not induce intracellular calcium mobilization (Supplementary Table S1). These data suggest that it was unlikely that calcium influx through voltage-dependent calcium channel affects the GABRP-induced metastatic potential in ovarian carcinoma cells.

### The *GABRP* promoter region was hypomethylated in metastatic tissues from ovarian cancer xenograft mice

**Figure 3:**
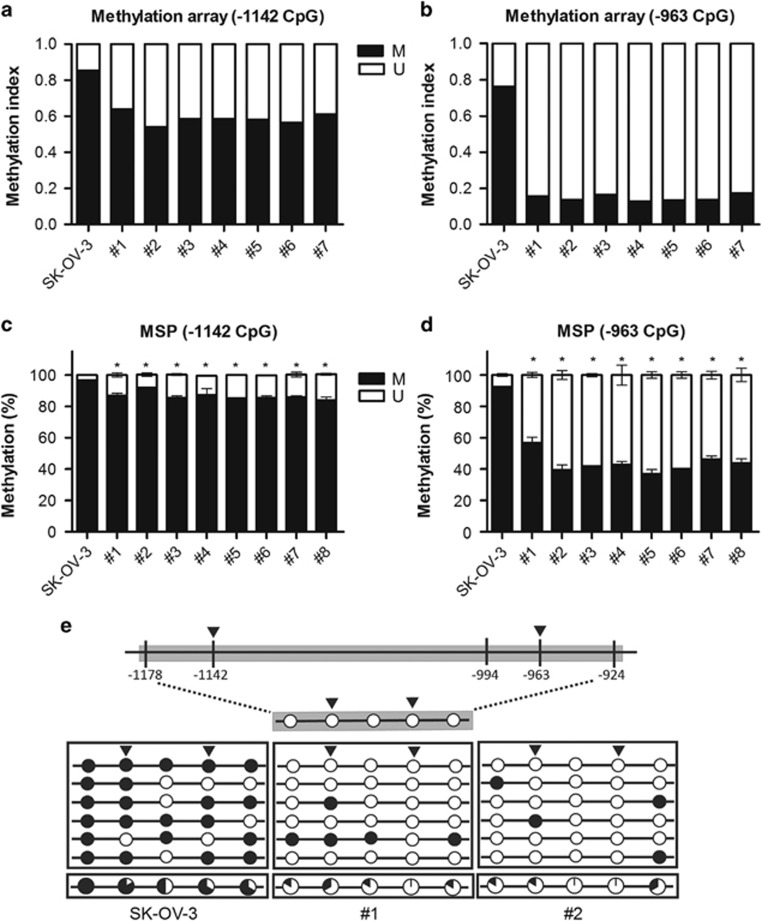
DNA methylation at the *GABRP* promoter CpG sites was altered in metastatic tissues from mouse xenografts. The DNA methylation status of *GABRP* at the −1142 and −963 CpG sites was analyzed using the Illumina HumanMethylation 450 BeadChip (**a**, **b**) and qMSP (**c**, **d**). The error bars represent the means±s.d.'s from three independent experiments. The DNA methylation status within the *GABRP* promoter region was analyzed by bisulfite sequencing (**e**). The *GABRP* promoter region is located between positions 170209520 and 170209971 of chromosome 5 in the human GRCh37/hg19 assembly and contains five CpG sites, which are located at −1178, −1142, −994, −963 and −924 from the transcription start site. Each circle represents a CpG dinucleotide. The methylation status of each CpG site is indicated with a black (methylated) or white (unmethylated) circle. The percentage of methylation at each site is indicated in a pie graph on the bottom line. The black segment of the pie graph is proportionally sized to represent the percentage of methylation. The triangles above the circles and bars indicate the CpG sites that were used for methylation array and qMSP analyses. The statistical analyses were performed using one-way ANOVA, followed by Bonferroni post tests (*P<0.05). M, the percentage of methylated CpGs; U, the percentage of unmethylated CpGs.

Previously, we performed genome-wide DNA methylation profiling using CpG methylation microarrays to examine the alterations in DNA methylation between metastatic tissues from ovarian cancer xenograft mice and ovarian carcinoma cells.^5, 6, 7^ Using these data, we identified two CpG sites within the *GABRP* promoter region that were clearly hypomethylated in all tested metastatic tissues compared with those of the ovarian carcinoma cells. DNA methylation at these two CpG sites, located at −1142 and −963 bp from the transcription start site, was decreased by 20% and 59%, respectively, in the metastatic tissues compared with those of the ovarian carcinoma cells (Figures 3a and b). Next, we performed qMSP and confirmed that DNA methylation at −1142 and −963 CpG sites was reduced approximately 5–13% and 36–56%, respectively, in the metastatic tissues compared with those of the SK-OV-3 cells (Figures 3c and d).

To further analyze the DNA methylation status of the *GABRP* promoter region, which is located at 170209520–170209971 on chromosome 5 and contains five CpG sites, we performed BSP analysis of DNA isolated from two representative metastatic tissues and from SK-OV-3 cells. All five *GABRP* promoter CpGs were hypomethylated in the metastatic tissues compared with those in the SK-OV-3 cells, and the largest decrease in DNA methylation was observed at the −963 CpG site (Figure 3e).

### *GABRP* expression was altered by DNA methylation-dependent epigenetic modifications

**Figure 4:**
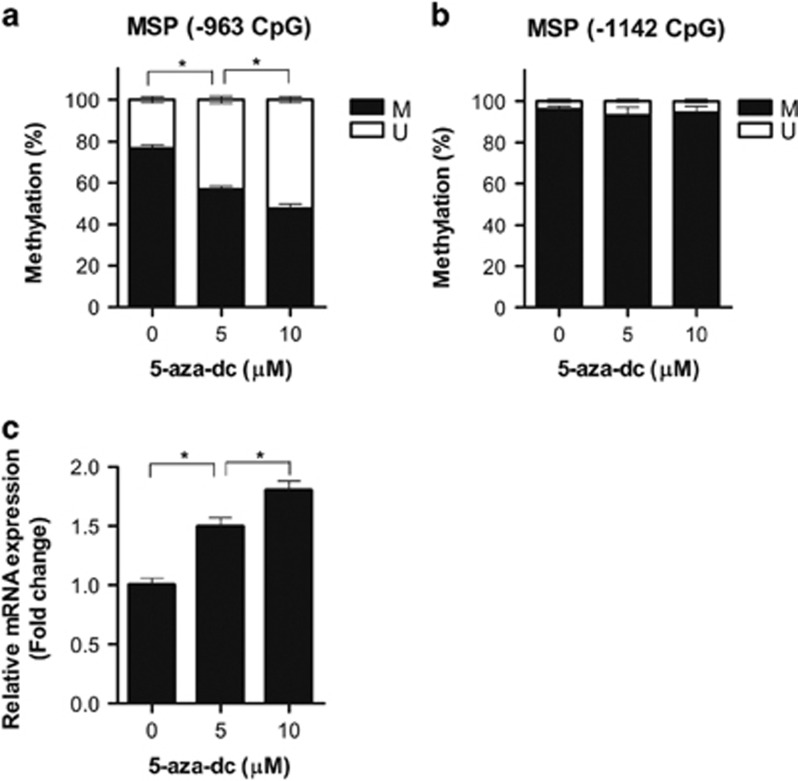
*GABRP* expression was regulated by DNA methylation-dependent epigenetic modifications. SK-OV-3 cells were treated with 0, 5 or 10 μM 5-aza-dc for 3 days. After 5-aza-dc treatment, the DNA methylation status at the −963 and −1142 CpG sites of the *GABRP* promoter was measured by qMSP (**a**, **b**), and *GABRP* mRNA expression was determined by RT-qPCR (**c**). The error bars denote s.d.'s of the means from three independent experiments. The statistical analyses were performed with one-way ANOVA and subsequent Bonferroni tests (*P<0.05). 5-aza-dc, 5-aza-2′-deoxycytidine; M, the percentage of methylated CpGs; U, the percentage of unmethylated CpGs.

To determine whether *GABRP* is regulated by DNA methylation-dependent epigenetic modifications, we treated SK-OV-3 cells with the DNA methyltransferase inhibitor 5-aza-dc and then measured the levels of DNA methylation at the *GABRP* promoter CpGs by qMSP and of *GABRP* mRNA expression using RT-qPCR. Treatment with 5-aza-dc resulted in a decrease in DNA methylation at the −963, but not the −1142, CpG site (Figures 4a and b). Importantly, *GABRP* expression was increased ~1.8-fold, and in a dose-dependent manner, compared with that of untreated cells (Figure 4c). These data indicate that *GABRP* expression is regulated by a DNA methylation-mediated epigenetic mechanism, and the DNA methylation status of the −963 CpG site is critical for the transcriptional regulation of *GABRP*.

### The *GABRP* promoter was hypomethylated in advanced stage, but not early-stage, primary ovarian tumors

**Figure 5:**
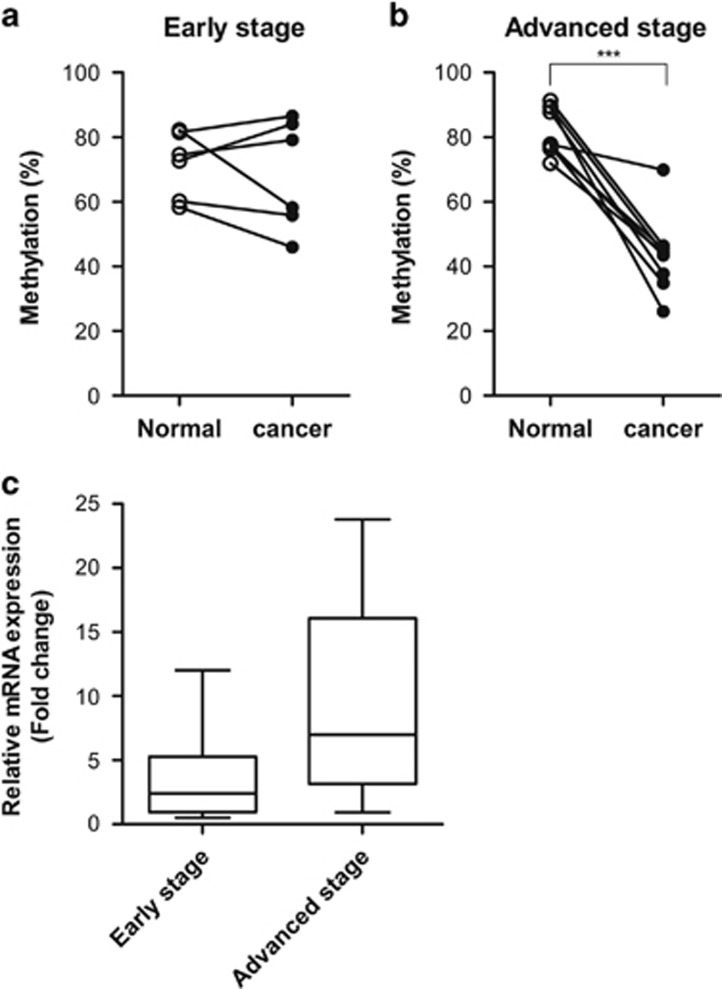
The *GABRP* promoter is hypomethylated in patients with advanced ovarian cancer. The alteration in DNA methylation levels at the −963 CpG site between paired primary tumor and adjacent normal tissues from individual patients with early-stage (**a**) or advanced stage (**b**) ovarian cancer was analyzed using qMSP. *GABRP* expression levels of primary tumors relative to those of paired normal tissues were measured by RT-qPCR (**c**). The data are represented by box-and-whisker plots from minimum to maximum values, and the lines inside the boxes represent the medians. The statistical analyses were performed using *t*-tests (***P<0.001). qMSP, quantitative methylation-specific PCR.

To further validate the clinical significance of our findings, we next investigated the DNA methylation status at the −963 CpG site within the *GABRP* promoter, and *GABRP* expression in 15 paired clinical samples, which included primary ovarian carcinomas and adjacent normal tissues, from six patients diagnosed at early stage and nine patients at advanced stage. Although the DNA methylation at the −963 CpG site of the *GABRP* promoter was not significantly altered in early-stage primary tumor tissues, it was reduced in advanced-stage primary tumor tissues, compared with that of normal tissues (Figures 5a and b). More specifically, 89% of the advanced-stage patients had DNA methylation levels at the −963 CpG site that were decreased by more than 20% in the tumor tissue relative to the paired normal tissue, whereas this decrease was only observed in 17% of the early-stage patients. Consistent with these data, the median value of relative *GABRP* expression was higher in advanced-stage tumor tissues than in early-stage tumor tissues, although, the data were not statistically significant (Figure 5c). To examine the association between the expression level of *GABRP* and aggressiveness of tumors in serous-type ovarian cancer, we performed microarray gene expression analysis using The Cancer Genome Atlas (TCGA) database, which include gene expression data from 138 patients with ovarian cancer and lymphatic invasion and 80 patients with ovarian cancer but without lymphatic invasion. Regardless of lymphatic invasion, the average *GABRP* expression was higher in the advanced stage than in the early-stage tumor tissue, but the differences were not significant in either subgroup, with lymphatic invasion (*P*=0.664) or without lymphatic invasion (*P*=0.303) (Supplementary Table S2). These findings suggest that aberrant demethylation of specific CpG sites within the *GABRP* promoter is associated with the aggressiveness of ovarian carcinoma cells.

## Discussion

Although it is well known that GABA and GABA receptors function as predominant inhibitory neurotransmitters in the mature mammalian brain, their role in non-neuronal cells, including cancer cells, is largely unknown. More recently, it was reported that GABAergic signaling is involved in the development of many tissues, including the peripheral nervous system, lung, liver, pancreas and testis,^19^ and that it regulates proliferation, migration and invasion in different cell types.^20^ Several studies have also shown that GABA receptors are involved in tumorigenesis, migration, invasion and proliferation of cancerous cells,^12, 15, 16, 19, 20, 21, 22^ however, the functions of GABA receptors in these processes seem to be cancer-type dependent. For instance, Chen *et al.*^20^ reported that GABA-induced cell migration and invasion are suppressed in HCC human liver cancer cells through the GABA_A_ receptor, whereas another study reported that the GABA_A_ receptor alpha3 promotes breast cell migration by activating the AKT pathway.^23^

The GABA receptor *GABRP* is overexpressed in pancreatic ductal adenocarcinoma cells^15^ and basal-like subtype of breast cancer,^16^ where it has been shown to promote proliferation, tumorigenesis and migration. Similarly, in this study, we showed that levels of *GABRP* were markedly upregulated in metastatic tissues from an ovarian cancer xenograft mouse model compared with those of SK-OV-3 cells. Furthermore, we performed *GABRP* gain- and loss-of-function studies in the SK-OV-3 ovarian carcinoma cell line to investigate the effect of GABRP on cell migration and invasion. The results showed that GABRP promoted cell migration and invasion of ovarian carcinoma cells, suggesting it has an essential role in the acquisition of an aggressive phenotype in ovarian cancer cells. Finally, treatment with the MEK inhibitor U0126 suppressed the migratory and invasive abilities of ovarian carcinoma cells, indicating that these functions of GABRP are mediated via ERK pathway activation. Our newly identified function and mechanism of GABRP in ovarian cancer cells are consistent with those shown previously in pancreatic and breast cancer cells.

To address the mechanism of GABRP overexpression in metastatic ovarian cancer cells, we analyzed genome-wide DNA methylation profiling data and identified two *GABRP* promoter CpG sites at −1142 and −963 bp from the transcription start site that were hypomethylated in metastatic tissues from xenograft mice compared with those in the injected ovarian carcinoma cells. We further showed that treatment with a DNA methyltransferase inhibitor recovered the suppressed expression of *GABRP*, indicating that *GABRP* expression is regulated by a DNA methylation-dependent epigenetic mechanism, which had not been previously shown. Our results also showed that the DNA methylation status at the −963 CpG site was critical for the transcriptional regulation of *GABRP*.

To demonstrate the clinical relevance of our findings, we showed that the DNA methylation at the −963 CpG site of *GABRP* was decreased in primary ovarian tumors of advanced stage, but not of early stage, compared with that of their paired normal tissues, strongly suggesting that hypomethylation at the −963 CpG site is a marker for an aggressive form of ovarian cancer. Although not statistically significant, we also observed higher expression of *GABRP* in several primary tumors of advanced stage than in primary tumors of early stage. Hence, we speculate that ovarian carcinoma cells with upregulated *GABRP* expression, due to the hypomethylation of specific *GABRP* promoter CpG sites, acquire aggressive phenotypes, which subsequently leads to their metastasis.

## Figures and Tables

**Table 1:** Clinicopathological characteristics of 15 ovarian carcinoma cases

*Patients no.*	*Age*	*Stage*^a^	*Grade*^b^	*Histology*
#1	57	IIIc	G3	Endometrioid adenocarcinoma
#2	53	IVa	G2	Serous adenocarcinoma
#3	64	IIIc	G3	Serous papillary carcinoma
#4	70	IIIc	G3	Serous papillary carcinoma
#5	48	IIIc	G3	Serous carcinoma
#6	51	IV	G3	Serous papillary carcinoma
#7	42	IVb	G3	Serous papillary carcinoma
#8	54	IIIc	G3	Serous carcinoma
#9	55	IIIc	G3	Serous carcinoma
#10	45	Ic1	G3	Clear cell carcinoma
#11	49	Ia	G2	Endometrioid adenocarcinoma
#12	54	Ic2	GX	Mucinous borderline tumor
#13	42	IIb	G3	Mixed endometrioid adenocarcinoma and serous adenocarcinoma
#14	38	IIa	GX	Clear cell carcinoma
#15	52	Ia	G2	Clear cell carcinoma

a International Federation of Gynecology and Obstetrics (FIGO) stage.

b Histological grade.
