# Supplementary figures and images for: Aberrant epigenetic regulation of GABRP associates with aggressive phenotype of ovarian cancer
Source: Exp Mol Med. 2017 May 19;49(5):e335–. doi: 10.1038/emm.2017.62 (PMC5454450; doi:10.1038/emm.2017.62)

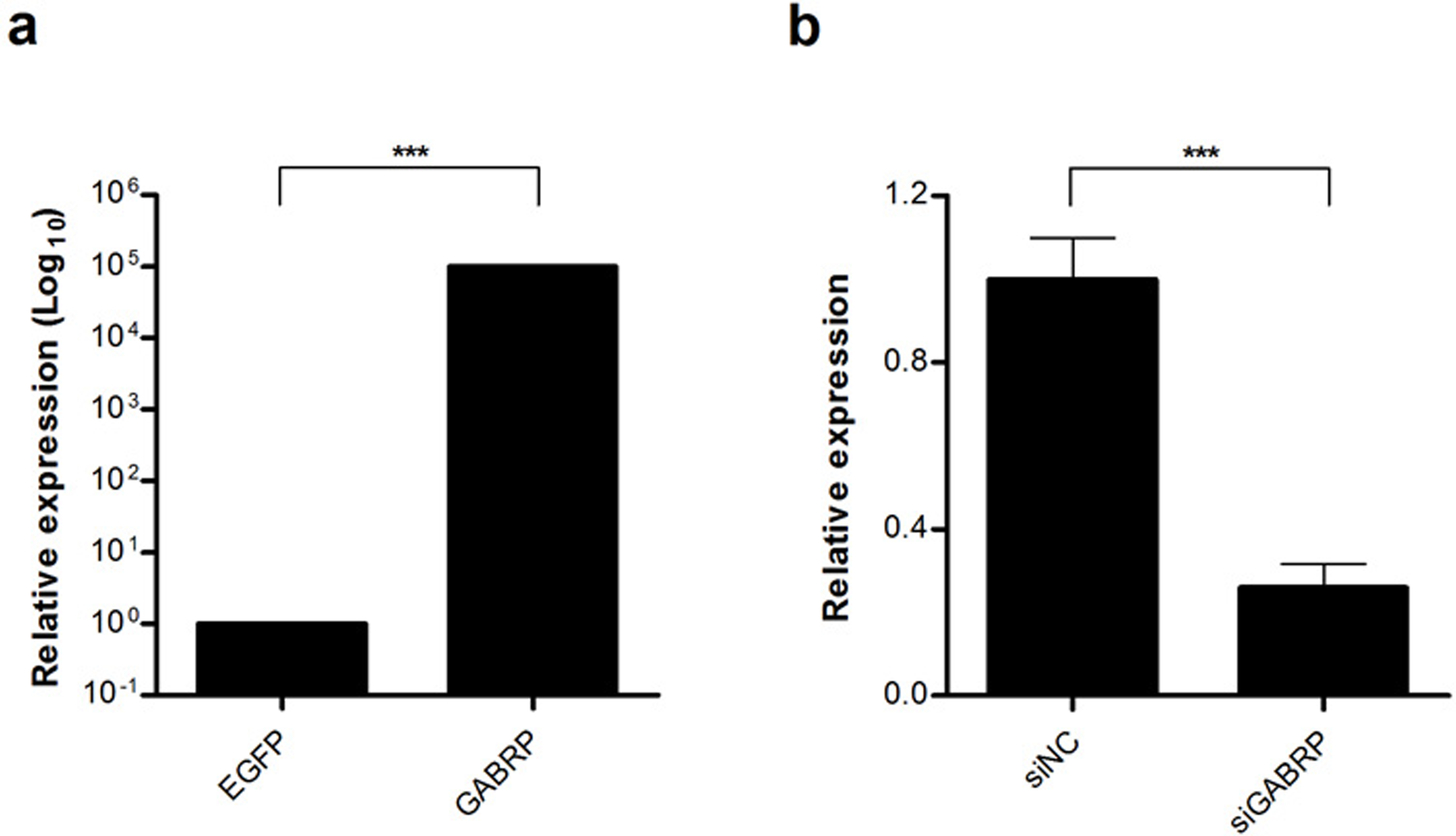

Supplement: Supplementary Figure S1 [file emm201762x1.tif]
