# Supplementary material for: Aberrant epigenetic regulation of GABRP associates with aggressive phenotype of ovarian cancer
Source: Exp Mol Med. 2017 May 19;49(5):e335–. doi: 10.1038/emm.2017.62 (PMC5454450; doi:10.1038/emm.2017.62)
Supplement: Supplementary Information [file emm201762x2.doc]

**supPLEMENTARY INFORMATION**

**supPLEMENTARY Method**

**Detection of calcium concentration in the cells**

SK-OV-3 cells were transfected with *EGFP* and *GABRP* expression constructs or siNC and siGABRP. After 24 h of transfection, the cells were treated with/without 100 µM of muscimol (Sigma-Aldrich, St. Louis, MO, USA) for 1 h. The calcium ion concentration was determined using the calcium detection kit (Abcam, Cambridge, UK) according to the manufacturer’s protocol. Transfected or treated SK-OV-3 cells (2 × 10^6^) were homogenized in 150 μl of calcium assay buffer and then centrifuged. Calcium concentrations in supernatant materials were measured by reading the absorbance at 575 nm using a microplate reader.

**GABRP expression analysis using public database**

Clinical and mRNA expression data related to the *GABRP* gene in The Cancer Genome Atlas (TCGA) ovarian cancer dataset were collected from the TCGA portal. We collected clinically annotated data from 573 serous ovarian cancer patients in TCGA datasets in which gene expression profiles were analyzed using an Affymetrix Human Genome U133 Array. Of these patients, 218 samples had lymphatic invasion and stage information annotations. Of these samples, 138 had lymphatic invasion and 80 did not have lymphatic invasion. We first measured 205044_at (*GABRP* gene) expression with and without lymphatic invasion to determine the difference in *GABRP* expression between early and advanced stages.

Data analyses were conducted using Bioconductor R 3.0.2^1^ and IBM SPSS statistics 23 (SPSS, Chicago, IL, USA). Background adjustment was carried out employing the Robust Multi-Array Analysis (RMA) algorithm from the Affy package for preprocessing. The independent samples *t*-test was used to compare average *GABRP* gene expression between the early stage (I, II) and advanced stage (III, IV).

**References**

1. Reimers M, Carey VJ. Bioconductor: an open source framework for bioinformatics and computational biology. *Methods Enzymol* 2006; **411**: 119-134.

**supPLEMENTARY Figure legend**

**Figure S1.** *GABRP* transient transfection efficiency was confirmed by measuring *GABRP* mRNA expression using RT-qPCR. SK-OV-3 cells were transfected with *EGFP* and *GABRP* expression constructs (a) or with siNC and siGABRP (b). The error bars indicate the mean ± SD. Statistical analyses were performed with *t*-tests (^***^p < 0.001).

**supPLEMENTARY TABLE**

**Table S1. The calcium concentrations (mM) of transfected or treated cells.**

|  | No treatment | 100 µM Muscimol |
| --- | --- | --- |
| Control | 0.24±0.040 | 0.47±0.014 |
| EGFP | 0.30±0.030 | 0.46±0.021 |
| GABRP | 0.30±0.013 | 0.48±0.037 |
| siNC | 0.25±0.016 | 0.47±0.058 |
| siGABRP | 0.25±0.026 | 0.49±0.067 |

EGFP, enhanced green fluorescent protein; GABRP, γ-aminobutyric acid (GABA)_A_ receptor π subunit; siNC, non-targeting control siRNA; siGABRP, *GABRP* siRNA.

**Table S2. *GABRP* in TCGA data for patients with ovarian cancer with or without lymphatic invasion**

| **Without** | **Patients** | **Average** | **With** | **Patients** | **Average** |
| --- | --- | --- | --- | --- | --- |
| **Lymphatic Invasion** |  | **Expression** | **Lymphatic Invasion** |  | **Expression** |
| Early stage | 15 | 4.013951 | Early stage | 12 | 4.111756 |
| Advanced stage | 65 | 4.190359 | Advanced stage | 126 | 4.244537 |
| Total | 80 | 4.157282 | Total | 138 | 4.23399 |
